# Incorporating Variable Porosity into the Determination of Effective Permeability in Interchanging Double Cloth Woven Fabrics Using Darcy’s Law

**DOI:** 10.3390/polym15143048

**Published:** 2023-07-14

**Authors:** Ana Kalazić, Tea Badrov, Ivana Schwarz, Snježana Brnada

**Affiliations:** Department of Textile Design and Management, Faculty of Textile Technology Zagreb, University of Zagreb, Prilaz baruna Filipovića 28a, 10000 Zagreb, Croatia; ana.kalazic@ttf.unizg.hr (A.K.); tea.badrov@ttf.unizg.hr (T.B.); ivana.schwarz@ttf.unizg.hr (I.S.)

**Keywords:** woven fabric, layer interchanging double cloth, air permeability, Darcy flux, volume porosity

## Abstract

Woven fabrics are widely used for thermal protection due to their porosity, which provides thermal insulation and breathability. This research focuses on investigating the influential parameters in the thermal protective properties of layer interchanging double cloth, including the woven structure and varying yarn fineness. The properties affecting the protective properties and comfort of multilayered woven fabrics include the fabric thickness, fabric porosity, and air permeability. Darcy’s law is applicable for determining the effective air permeability of woven fabrics. By understanding and controlling fabric porosity, it becomes possible to develop thermal protective clothing that combines improved comfort, cost-efficiency, and effectiveness. This study represents a novel approach for the clarification of airflow permeability behavior in complex structures of elastic multilayer woven fabrics using Darcy’s law. This innovative approach expands the understanding of permeability in fabrics beyond single-layer fabrics with vertical pores or 3D fabrics used in resin injection processes.

## 1. Introduction

Woven fabrics are highly porous textile materials made by interlacing warp and weft yarns in a certain pattern. This structure makes the fabric a very heterogeneous planar material in which the volume units of air between the warp and weft yarns make the material porous. The porosity of the fabric indicates the share of pores in the fabric.

When describing the properties and behavior of woven fabric, it can be viewed as a two-dimensional or three-dimensional material. Accordingly, there are two types of pores in woven fabric: vertical (open) and volume pores. Porosity is the ratio of the total amount of empty space in the material to the largest volume (domain) occupied by the material. This property is essential for the thermal insulation properties of woven fabrics for technical protective purposes such as firefighting. Air trapped in the pockets of woven fabric pores, as a good heat insulator, will increase heat protection, and a porous structure will increase the breathability of the fabric. Porosity is directly related to the permeability of fluids, air, and light through the woven fabric, which is influenced by the thickness of the fabric, the shape and size of the pores, and the distribution of the space between the yarns. The problem with calculating the woven fabric permeability is the non-uniformity in the distribution of the pores, the spacing between the yarns, as well as their irregular undefined shapes, and the non-uniform thickness of the woven fabric.

During the work process, in emergency interventions, the human body is exposed to various dangers. The choice of raw materials in the design process of protective woven fabrics has to contribute to the most effective protection and at the same time ensure comfort [1]. Technological development, as well as the invention of synthetic fibers, has significantly changed the development and production of protective woven fabrics for targeted applications. The demands and high standards placed on textile materials for thermal protective clothing have led to innovations in the development and production of fabrics.

Protective clothing for firefighters is necessary when extinguishing fires and engaging in similar activities that involve the danger of direct flames and high levels of thermal stress [2]. Such clothing must be selected based on risk assessment and must provide protection from other extreme conditions such as protection from cold, rain, and water, for extinguishing, mechanical actions, aggressive and reactive chemicals, hazardous chemicals, including chemical solutions, etc. General requirements that protective clothing for firefighters must meet include the requirement for thermal protection, comfort during interventions, comfort when being worn in normal climatic conditions, and a suitably designed ventilation system to release heat produced by the natural metabolism of the firefighter. An important requirement for protective clothing for firefighters is comfort, which can be divided into thermophysiological comfort, sensory comfort, and comfort while using/wearing the clothing. Protective firefighter clothing protects the upper body, neck, arms, and legs, with no protection for the head, hands, and feet. It can be divided into firefighter workwear, firefighter dress uniforms, firefighter intervention clothing, and other equipment.

The development of fabrics that can efficiently protect the body requires careful attention being paid to functionality and effectiveness. This could be achieved by designing complex woven fabrics known as layer interchanging double cloth, which use fibers of different raw material compositions with integrated protection properties. Such woven fabrics offer multi-functionality, meeting mechanical, thermal, and thermo-physiological requirements in a single process that is cost-efficient and environmentally friendly. However, layer interchanging double cloth is a complex structure and requires extensive knowledge for woven fabric development, production, and application. This woven fabric is created by interlacing two layers of fabric on one loom, using warp or weft threads. The interlacing threads may differ from other threads in terms of raw material, fineness, and production technology. It is essential to coordinate the weave of the upper and lower layer to ensure that the interlacing threads are not visible on either side. Layer interchanging double cloth has the potential to be a game-changer in the development of protective clothing due to its multi-functional properties, but it is not as widely used as conventional single-layer woven fabrics.

This research aims to investigate the influential parameters on the multifunctional properties of thermal protective layer interchanging double cloth, such as the woven structure and varying yarn fineness. Compared to current multi-layer protective clothing, layer interchanging double cloth is more cost-efficient and eco-friendly, making it a promising material for the development of effective thermal protective clothing with increased comfort at a lower cost.

## 2. Properties Affecting the Protective and Comfort Properties of Multilayered Woven Fabrics

Multilayered woven fabrics consist of at least two yarn systems in one direction and one yarn system perpendicular to it. Their characteristic is an increased thickness compared to simple structures and better thermal insulation properties. By using complex structures, it is possible to design a woven fabric with different expected properties on the front and back, with the domination of one fiber composition on the front and the other on the back. In two weft systems, the upper weft is on the front side, and the lower weft is on the back side (if a thicker fabric is to be made, a third-middle weft or a special warp is used). By changing the weave, various effects can be achieved, such as stripes, squares, etc. By carefully designing the structure of multi-layered woven fabrics, it is possible to obtain optimal protection and comfort properties of the fabric by finding the optimal ratio of the thickness and porosity parameters [3].

### 2.1. Fabric Thickness

Fabric thickness has an important role when it comes to the comfort, thermal insulation properties, behavior, and end-use of textiles. It is the largest distance between the highest peaks on the front and the back of the woven fabric and is measured in millimeters. The change in fabric thickness depends on the individual preparatory stages of the weaving process. In addition, it depends on various factors that affect the shaping of the fabric during the weaving process and during the finishing processes [4]. Fabric thickness (t) is a third dimension that is significantly smaller than the width and length of the fabric, but at the same time, it is crucial because it affects the performance and the main characteristics of the woven fabric. The thickness of the woven fabric depends on the diameter of the warp and weft yarns, warp and weft densities, type of weave (construction), raw material composition, thread tension during weaving, and the type of finishing. The thickness of the fabric must be measured under the minimum pressure of the measuring device.

### 2.2. Fabric Porosity

Fabric porosity is directly related to the permeability of fluid and air (gas) through the fabric, which is affected by the fabric thickness, the shape and size of the pores, as well as the distribution of the space between the yarns. Fabric porosity can be observed on several levels [5]:

- The porosity between the fibers;
- The porosity between the yarns;
- The effective porosity of the airflow, which is described as the function of fiber porosity and the porosity of the yarn itself.

Fabric porosity is an important parameter in evaluating the comfort of clothing and the physical characteristics of technical textiles [6]. The fabric pore properties, such as pore size, pore size distribution, pore shape, and porosity, are determined by the yarn and fiber properties as well as the fabric’s structural properties, such as weave, warp, and weft density, thickness, etc. Thus, predicting the air permeability of the fabric used in a certain area can be regulated by controlling the properties of the pores, which are determined by the structural properties of the fabric. The fabric structure in plain weave is relatively easy to describe. Fabrics in other weaves (for example twill or satin) are more complex because the weave of the fabric is a parameter that greatly affects the air permeability value of the fabric. It was found that the differences in air permeability between different types of fabric weaves are more noticeable in fabric samples with lower warp and weft density, while in fabrics with higher warp and weft density, this difference is less noticeable.

In general, all spaces that are filled with air can be considered pores in a woven fabric. However, it only indicates how much air is contained in the fabric and does not say anything about the shape of the pores, their size, or distribution. These characteristics are very important in terms of fabric air permeability [5].

It should be noted that the yarn diameter value is used in numerous models that describe fabric porosity. The cross-sectional shape of the yarn is variable along its length. The spatial geometry of the fabric is generally influenced by the type of weaving, yarn composition and yarn fineness, the structural parameters, and the type and adjustment of the loom. The problem of determining the yarn diameter can be further complicated because of the yarn’s hairiness and high unevenness.

There are two types of woven fabric porosity—vertical and volume porosity.

Vertical porosity denotes the percentage of the surface area of macro-pores in a unit area of fabric. It is calculated based on the cover factor of the woven fabric. To describe the vertical porosity, one can consider the elliptical vertical pore model, which is also a two-dimensional porosity model, but it is a projection of the fabric onto the vertical plane. Since warp density is usually greater than weft density, an elliptical pore cross-sectional shape is used to represent the situation. The following parameters are commonly used to compare porosity fabrics: pore cross-sectional area, pore surface distribution, pore density, equivalent pore diameter, maximum and minimum pore diameter, pore length, pore volume and open area fraction, pore volume fraction, etc. [7].

Volume porosity denotes the percentage of the sum of the pores’ volume in the total volume of the fabric (Equation (1)). Volume porosity takes place when the woven fabrics are viewed as a three-dimensional formation. Void spaces (pores) can be found in fibers, between the fibers in the yarn, and between the warp and weft threads in the fabric. As textile materials, woven fabrics have, compared to knitted fabrics or non-woven textiles, the most precisely determined internal geometric model of the porous structure in the form of a tubular system, where each macro-pore has a cylindrical shape with a permanent cross-section of their entire length [8].

(1) $$\varepsilon =\frac{V_{Y}}{V_{F}}\cdot 100$$

where ε is the total porosity in %, V_Y_ is the volume of yarn, and V_F_ is the volume of fabric (domain), and it is expanded:

(2) $$\varepsilon =1-\frac{m[g/m^{2}]}{L[mm]\cdot 1000\cdot \rho_{f}[\frac{g}{cm³}]}$$

where *m* is the fabric mass per unit area (g/m^2^), *L* is the thickness of the woven fabric (mm), and *ρ* is the fiber density (g/cm^3^).

### 2.3. Air Permeability

Air permeability is one of the most important properties of textile materials. It is generally expressed as the ability of an air-permeable fabric to transmit air under certain conditions. When it comes to clothing materials, permeability is an important aspect of comfort. In the case of technical textiles, permeability can even be a property essential to the function of the fabric (e.g., filters, parachutes, and airbags). The air permeability of woven fabrics is the main property of the internal textile material structure. A very small change in the fabric structure causes a change in its permeability. The size of pores in textiles, as well as their arrangement and distribution of shapes, are crucial characteristics of the fabric from the point of view of air permeability.

There is an assumption that if the pores between the yarns are large enough and the air has enough space for free passage, it will flow mostly that way. Therefore, in terms of air permeability assessment, the porosity within the yarn is usually neglected [9].

## 3. Applicability of Darcy’s Law in Textiles

### 3.1. Darcy’s Theory

Henry Darcy published his findings of an experiment he conducted to examine the movement of water through a sand bed, in 1856. This experiment led to the development of a mathematical principle that explains how fluids behave in such porous materials. According to Darcy’s Law, the potential energy gradient inside a fluid is proportional to the rate of flow through a porous media [10,11]. Darcy’s law is commonly used in the field of fluid mechanics, specifically in the study of porous media flow. It has wide applications in various disciplines, including hydrogeology, civil and environmental engineering, petroleum engineering, soil science and agricultural engineering, and geosciences [12].

From then until today, his theory has been developed in the above-mentioned areas. In the mid-20th century, there was a growing interest in utilizing Darcy’s law to study nonhomogeneous materials characterized by nonuniform and rapidly changing airflow velocities. The generalized law is then used to solve a few cases of flow near the boundaries of porous media [13].

In its most basic form, Darcy’s law says that the discharge rate q is proportional to the gradient in the hydraulic head and the hydraulic conductivity:

(3) $$q=\frac{Q}{A}=-K\frac{dh}{dl}$$

where q = Q/A is the specific discharge [L/T], A = cross-sectional area, and K is the hydraulic conductivity [L/T].

### 3.2. Literature Review

Different authors have taken Darcy’s law into account in their research in the field of textiles. Darcy’s law is commonly used in the textile industry, especially for nonwoven textiles with diverse pore structures. Early research [14] showed that laminar flow models did not match experimental data, leading to the use of Darcy’s law for approximate permeability values. Later studies [15,16] found a strong correlation between air and water permeabilities in fabrics, influenced by fabric porosity. Resin impregnation in nonwoven textiles followed Darcy’s law, with variations in impregnation rates between macropores and micropores [17]. Geotextiles, subjected to long-term compressive loads, were studied [18], and modified Darcy models were developed [19]. Recently, various 3D numerical simulation models have been used in applications such as filtration [20] and determining permeability correlations with other parameters in industrial applications [21].

In the literature, there are numerous studies focusing on the air permeability of fabrics, with most of them concentrating on single-layer fabrics with dominant vertical porosity where laminar flow laws can be applied. Kulichenko and Langenhove [22] established a predictive equation using the Poiseuille formula and Darcy’s law. While this equation theoretically analyzes the relationship between permeability and fabric structure, it is limited to open fabrics with vertical porosity and lacks experimental validation. Gooijer and Warmoeskerken [23] developed a geometrical model that allows the application of theoretical models to densely woven fabrics, specifically filament fabrics. However, their model does not consider the interstices between the fibers. Chen et al. [24] predicted permeability in mesopores through numerical simulation and used Gebart’s analytical model [25] for micropores. They then deduced in-plane permeability based on Darcy’s law under specified boundary conditions. Their results showed good agreement with experimental data for three plain weave fabrics available in the literature [26]. Dimitrovski et al. in their research [27] describe the porosity or inner porous structure of woven fabrics as a complex process that involves analyzing various porosity parameters such as the size, number, and distribution of pores. They developed a new method to determine the equivalent average size of pores in woven fabrics. The authors investigated exclusively vertical pores of assumed cylindrical shape that allow the same air permeability as the real woven sample with the same number of pores. The theory of fluids flow for laminar flow through circular tubes in the range of laminar flow was applied by the Hagen Poiseuille equation which was used to determine the average diameter of vertical pores and to describe the passage of air through the textile material. Using a newly developed method for determining the equivalent pore diameter, they obtained more accurate values of the average vertical pore diameter and a more precise description of the fabric’s internal structure by measuring the air permeability at different pressure drops.

While Poiseuille’s law needs precise knowledge about the flow geometry, such as the tube’s radius, Darcy’s law just needs the hydraulic conductivity, an empirical constant. As long as the flow is laminar, Darcy’s law can be applied to almost any porous medium. Considering the complexity of the structure of multilayer interchanging double cloth, in this paper, Darcy’s theory was used to determine the effective permeability of the material and assess the state of the structure.

Some assumptions need to be considered when using Darcy’s law. Firstly, the saturating air must be inert and not interact with the porous medium—the woven fabric. Secondly, the flow through the sample must be laminar. Thirdly, the airflow should be single-phase and completely saturate the porous medium. Lastly, the permeability of the medium should be constant and not vary with the nature of the fluid, flow rate, or pressure.

In the field of permeability in multilayer woven fabrics, most studies focus on applying Darcy’s law to predict the resin flow behavior through complex structures of engineered fabrics. In the book chapter [28], a comprehensive study on the permeability modeling of multilayer woven fabrics is presented. The chapter describes a framework for flow permeability measurement in resin transfer molding, where Darcy’s law is utilized to model the flow through the reinforcement fibers, with permeability serving as a measure of the fibers’ resistance to flow. In their research [29], the authors investigate the permeability of layered fabrics by characterizing both macro-permeability and micro-meso-permeability (dual level) horizontally and vertically through the woven fabrics. Ansys-Fluent software was employed to simulate permeability, using Stokes–Brinkman equations to model flow in open (inter-fiber) and porous (within-fiber) regions based on Darcy’s law. The study highlights the importance of incorporating dual-level permeability into woven composites. In the study [30], ultrasound scanning in C-scan mode was employed to measure resin flow through the thickness of individual layers of carbon fiber woven fabric. The fabric’s permeability was calculated using Darcy’s law, and the results demonstrated that ultrasound scanning in C-scan mode is an effective method for measuring resin flow through the z-coordinate of a single fabric layer. In an experimental study conducted by Karaki [31], a method was developed for non-crimped stitched fabrics and 3D orthogonal fabrics. This method involved injecting the test fluid at alternating velocities, measuring the pressure by using a data acquisition unit at each velocity, and evaluating the transversal permeability based on Darcy’s law. This approach showed an improvement by providing multiple-point measurements for each permeability value instead of a single point. According to Xueliang Xiao [32], while Darcy’s law is applicable for analyzing liquid flow through fabrics, it is necessary to consider non-Darcy behavior to better describe the actual flow in fabrics. In a research study [33], an analytical model based on Darcy’s law was developed, which can be successfully used to predict the permeability of woven fabrics through their thickness under the influence of increasing air pressure. In Khan’s research [34], the resin flow through a fibrous reinforcement is governed by Darcy’s law, which states that the fluid flow rate is proportional to the pressure gradient. Dei Sommi et al. developed a straightforward way to calculate both in-plane and out-of-plane saturated permeability K_sat_ which involves measuring the volumetric flow rate, which can be used with Darcy’s law to determine K_sat_ [35]. This method is commonly used for calculating out-of-plane saturated permeability K_3-sat_ and does not require any specialized equipment or expertise beyond a flow meter and pressure gauge.

From the literature review, it is evident that a significant portion of research on air/fluid permeability through the thickness of fabrics is focused on single-layer fabrics with vertical pores or complex 3D fabrics used for reinforcement in resin injection processes. This study, however, represents the first investigation of airflow behavior in complex structures of multilayer fabrics using Darcy’s law. The application of Darcy’s law in this context is valuable for assessing the breathability of woven fabrics used for thermal protection, which is a crucial aspect often overlooked when considering the protection of individuals exposed to thermal hazards.

### 3.3. Applicability of Darcy’s Law in Determining Air Permeability Characteristics for Multilayered Woven Fabrics

The air permeability of a woven fabric refers to its ability to allow air to pass through its surface. Air permeability is quantified as the volume of air in milliliters that passes through 100 mm^2^ of fabric in one second. The reciprocal of air permeability, known as air resistance, is defined as the time in seconds for 1 mL of air to pass through 100 mm^2^ of fabric.

Darcy’s law is an equation that describes the flow of fluid through a porous medium. In cases where velocities are high and viscous effects are small compared to inertial effects, non-Darcian flow can occur. The Forchheimer equation is used to approximate the transitional region between Darcian and non-Darcian flow. Due to the complex and computationally expensive nature of pore structure characterization, macroscopic approaches are often employed as suitable approximations for Darcian and non-Darcian flow at different pressure and velocity ranges. Darcy’s law is applicable for low velocities and small pressure drops.

In its simplest form, the mathematical expression of Darcy’s law is as follows [36]:

(4) $$Q=\frac{-kA(p_{1}-p_{2})}{\mu L}$$

where Q—total discharge [m^3^/s], k—coefficient of permeability [m^2^], A—cross-sectional area of woven fabric exposed to airflow [m^2^], p_1_—initial pressure [Pa], p_2_—final pressure [Pa], μ—air viscosity 1.6 × 10^−5^ [kg/(m·s)], and L—fabric thickness [m].

Q [m^3^/s] is a volumetric flow rate, that is, the volume of air [m^3^] that passed through the surface of the fabric in 1 s.

Specific discharge (Darcy flux) is often referred to as the Darcy flux and it is not the velocity that the fluid traveling through the pores is experiencing [37]:

(5) $$q=-\frac{K}{\mu }∆p$$

where q—specific discharge [m/s] and ∆p—pressure drop p_1_–p_2_.

The fluid velocity (v) is related to the Darcy flux (q) by the porosity (ε). The flux is divided by porosity to account for the fact that only a fraction of the total formation volume is available for flow [37]:

(6) $$v=\frac{q}{\varepsilon }$$

The concept of effective porosity is employed to describe the portion of pores in a complex woven structure that is made unavailable for flow. The Darcy velocity, also known as specific discharge, is obtained by dividing the volumetric flux density (Q) by the bulk cross-sectional area (A) of the material. This measurement indicates the velocity of air passing through the woven fabric’s accessible pores.

**Figure 1:**
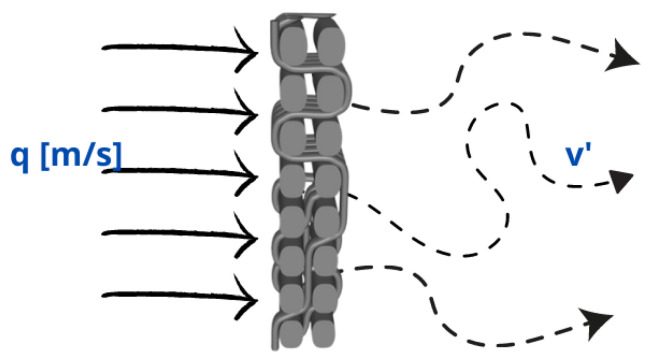
Air velocity throughout the porous two-layer woven composite space.

The actual air velocity varies throughout the porous space (Figure 1), due to the connectivity and geometric complexity of that space. This variable velocity can be characterized by its mean value. The average air velocity depends on how much of the cross-sectional area A is made up of pores and how the pore space is connected within the complex woven structure. The resultant spatial air velocity of air through the textile material will be reduced to the average of all local velocities v′.

## 4. Experimental Part

### 4.1. Development of Woven Fabric with an Increased Proportion of Pores with Improved Thermal Protection Properties and Comfort—Theoretical Assumptions

In order to increase thermal protection and comfort, a woven structure of the layer interchanging double cloth was developed.

**Figure 2:**
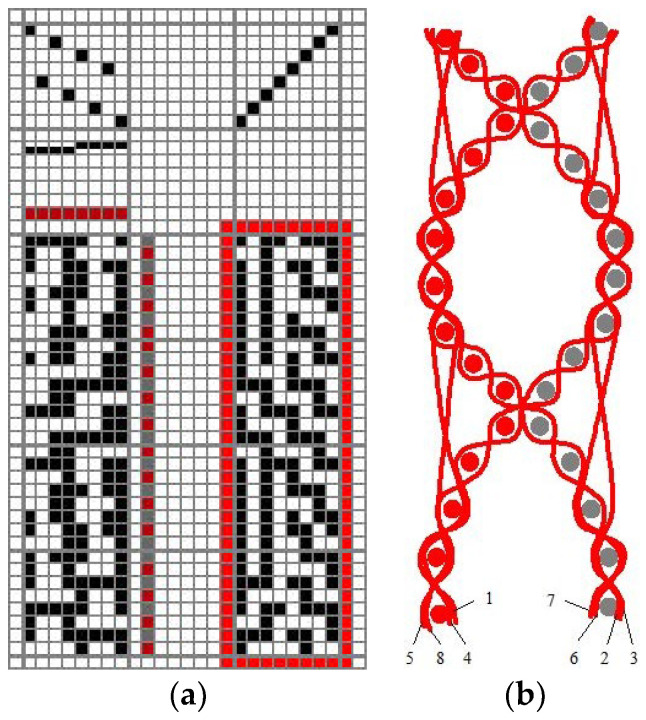
Structure of the layer interchanging double cloth [37]: (**a**) fabric weave and (**b**) fabric cross-section.

In the woven fabric cross-section (Figure 2b) it is visible that the fabric is structured in such a way that two layers form tubular cavities across the width of the fabric, that is, volume pores. The fabric weave unit consists of 8 different interlacement warp threads and 32 wefts, half of which are placed on the front and half of which are placed on the back of the fabric, which is regulated by programming the weft selector. The result is a layer interchanging double cloth with an increased proportion of volume pores. The layer interchanging double cloth can be described as a system of two plain weave fabrics periodically connected across the width, whereby the structure of the system of longitudinally connected tubular formations is obtained.

The choice of raw materials for the weft—aramid and modacrylic yarns—and their controlled change resulted in a complete aramid surface on the front of the fabric, while the reverse side is dominated by modacrylic yarns that ensure comfort on the back (next to the skin). This construction ensures satisfactory thermal protective properties [37] and high wearing comfort at the same time.

Considering the very complex structure of this material, it is not possible to describe the morphological characteristics of the pores in a simple way, especially considering the fact that the pore system is expectedly very heterogeneous, and their mutual connection is difficult to define. In the research of Badrov et al. [37], breathability is described through the water vapor resistance parameter, which can be related to the situation of the release of gaseous sweat from the microclimate (between the body and cloth layer) into the environment. In this paper, breathability will be described through the air permeability through the surface of the textile material, using Darcy’s theory to estimate the effective porosity expressed in the proportion of void volumes and also to estimate the stability of the porous structure with regard to the raw material used.

To establish the specific discharge (air permeability) and the structural stability of the porous layer interchanging double cloth, it is possible to apply the fluid theory, i.e., fluid flow through porous materials. The structure of the layer interchanging double cloth can be described as the system of porous zones of low and high flow velocities and areas where flow does not occur. Cross-section porosity, pressure drop, and superficial velocity have been identified as important values for characterizing flow/porous material air breathability.

### 4.2. Materials and Methods

To achieve specific protective properties in the woven fabrics, a careful selection of fibers was made. Aramid fibers, a mixture of meta and para-aramid fibers, were chosen because of their excellent mechanical properties, fire resistance, and thermal stability. Meta-aramid fibers are particularly successful in fireproof clothing as they do not ignite, melt, or drip and they maintain their mechanical properties at high temperatures. By combining them with para-aramid fibers, their breaking strength increases. For achieving better comfort and softness, a blend of flame-retardant modacrylic and cotton fibers was used. Modacrylic fibers can be blended with natural fibers. Because of their ability to remove oxygen from non-flame-retardant fibers, they can protect both themselves and the other fibers in the blend from burning and overheating. Cotton fibers were added for their comfort qualities. Table 1 shows the parameters of the used yarns.

Properties like fabric thickness, mass, vapor permeability, and thermal insulation are affected by the fabric weave and yarn fineness. The woven fabric samples used in this study are layer interchanging double cloth, which has a complex structure and is known for its vapor permeability and thermal insulation properties. For this study eight fabrics were woven, on the laboratory weaving machine DW598 (Fanyuan Instrument-FYI, Hefei, China), using the same weave and same yarn types as mentioned in Table 1, by varying the weft thread fineness while keeping the warp thread fineness constant. Specifications of the woven fabric samples are shown in Table 2.

The sample peg plan and the fabric cross-section are shown in Figure 2a.

**Figure 3:**
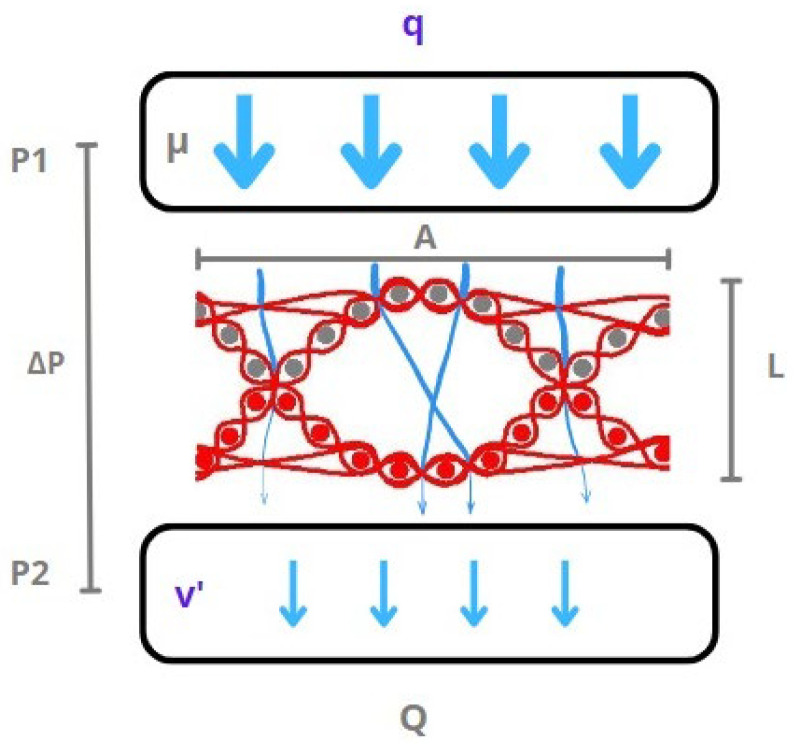
A schematic representation of an air permeability test of layer interchanging double cloth.

The woven fabric samples were tested for their air permeability using an air permeability testing device, Mesdan Air tronic plus. The air permeability (Figure 3) was tested at five different drop pressures, ranging from 5 Pa to 25 Pa, with regular increments of 5 Pa. The air permeability was tested by supplying a laminar airflow through the inlet tube of the device, gradually increasing the airflow volume until the desired drop pressure was achieved. The result of each test point is the flux discharge, q, measured in cm/s.

The fabric samples were tested from the front side (aramid weft) and the back side (modacrylic weft) under the following conditions:

- Testing area: 100 cm²
- Air volume: 10 L
- Pressure drop: 5, 10, 15, 20, and 25 Pa

The result of the testing process is the specific discharge, q, measured in cm/s, at each of the mentioned pressure levels.

The basic parameters of the woven fabric samples were tested according to the standards: fabric density—ISO 7211-5:2020; fabric thickness—ISO 5084:1997 (under a load of 1 kPa); and fabric mass per unit area—ISO 3801:1977.

## 5. Results and Discussion

### 5.1. Basic Parameters

The basic parameters of the tested woven fabrics are shown in Table 3. It can be observed that the measured densities of warp and weft do not significantly differ among the different samples. The mass and thickness values increase as the fineness values of the weft yarns increase. Based on the results of the basic parameters, it was determined that comparisons of the porosity among the different samples are possible in terms of considering them solely as a result of changes in the fineness of the weft yarn on the front and back of the fabric.

### 5.2. Fabric Volume Porosity

The volume porosity of the woven fabric samples was calculated using the fabric’s mass and thickness and the average fiber density in the fabric structure (Equation (2)).

**Figure 4:**
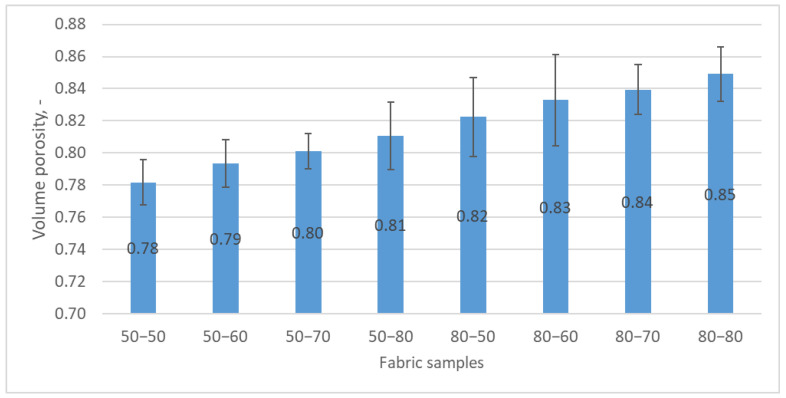
Change in the volume porosity of the woven fabrics with respect to the weft fineness.

From the graphical representation in Figure 4, it is evident that the volume porosity of the fabric increases as the fineness value of the yarn decreases, assuming that all other structural parameters of the fabric remain relatively unchanged.

### 5.3. Air Permeability of the Layer Interchanging Double Cloth

**Figure 5:**
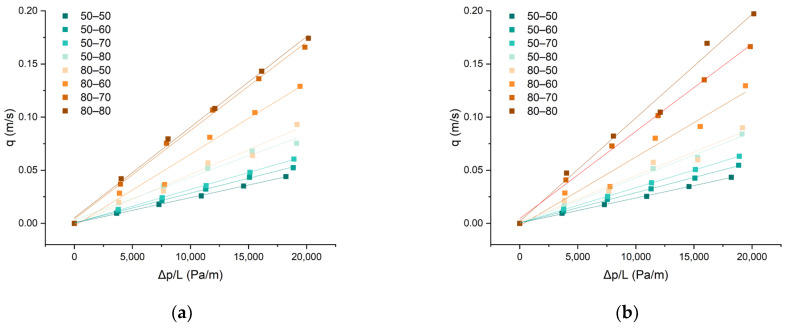
Dynamic air permeability: (**a**) front (aramid) side of the fabric and (**b**) back (modacrylic) side of the fabric.

In Figure 5, a plot of flow velocity (Q/A = q) against the pressure drop per unit length (∆p/L) on the woven fabric samples is presented. The air flux (q) values are linearly related to a slope equal to k/µ. The slope is determined for each approximate direction that corresponds to K air conductivity, i.e., air permeability.

**Figure 6:**
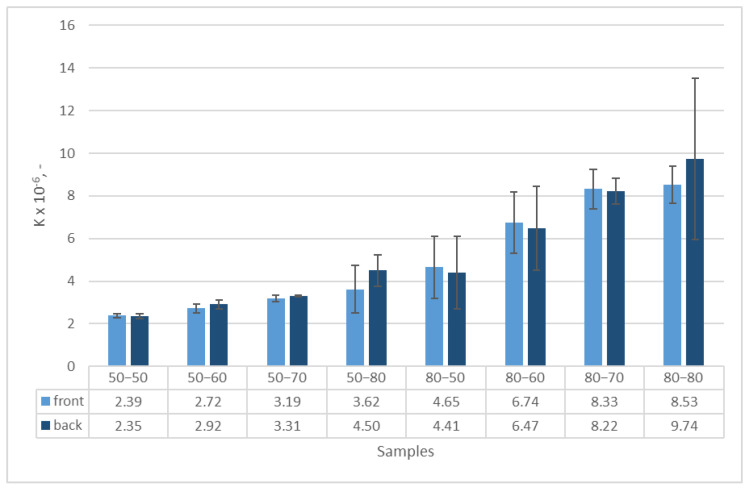
Permeability coefficient depending on the direction of airflow.

From the graphical representation in Figure 5, it is evident that there is a correlation between the fineness of the weft yarns and the air permeability of the fabric. Fabrics with lower average weft yarn fineness values exhibit a higher slope K, indicating greater air permeability on both the face and back of the fabric. The differences in permeability between the front and back of the same fabric samples were not significant (shown in the bar graph in Figure 6), indicating that the composition and position of the layer in relation to the direction of the airflow do not significantly affect permeability. From all of the graphical representations, it is clear that the air permeability property undergoes significant changes in the fabric samples, with a single layer composed of high-fineness yarns. Increasing the fineness in the second layer leads to larger jumps in differences in air permeability.

**Figure 7:**
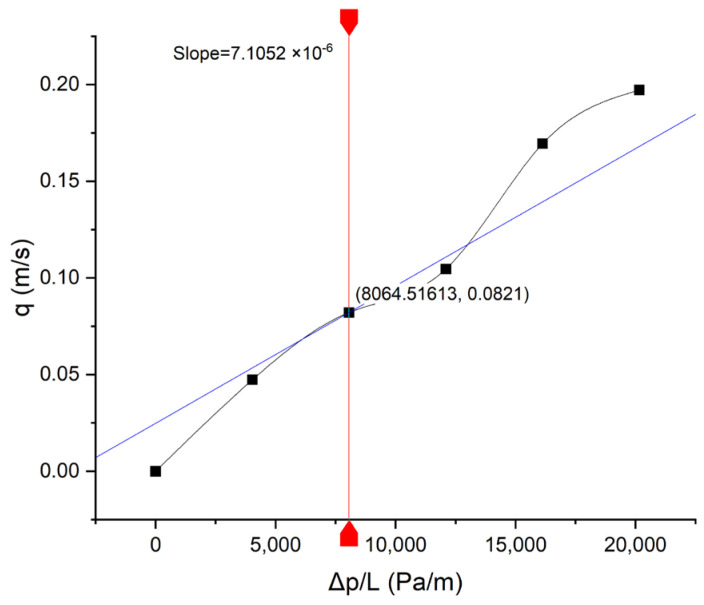
Air permeability tangent slope illustration.

Taking into account that the woven fabric samples are two-layered composites, with airflow passing through two textile barriers and being retained within a voluminous pore that extends across the entire width of the fabric, the nonlinearity of air permeability can occur. From the results, it is evident that more pronounced nonlinearity occurs in samples with larger differences in yarn fineness between the upper and lower fabric layers. This is due to the varying capacity of each layer to allow for air passage and the limited volume between these layers. When the airflow passes through such a complex textile composite with voluminous pores of irregular shapes and different dimensions within the structure, the laminar airflow no longer moves in a straight line but takes on different speeds depending on the pore shapes. Although, according to fluid theory, it is still considered laminar flow (Reynolds number < 2000), air permeability is no longer linear and can be described at each pressure drop as the current air permeability, or permeability at a specific pressure drop, as the slope of the tangent to the approximated curve through the points on the q∆p graph (Figure 7).

Turbulence is difficult to achieve in a porous medium. Velocity fluctuations dissipate through viscous interactions with the prevalent pore walls.

**Figure 8:**
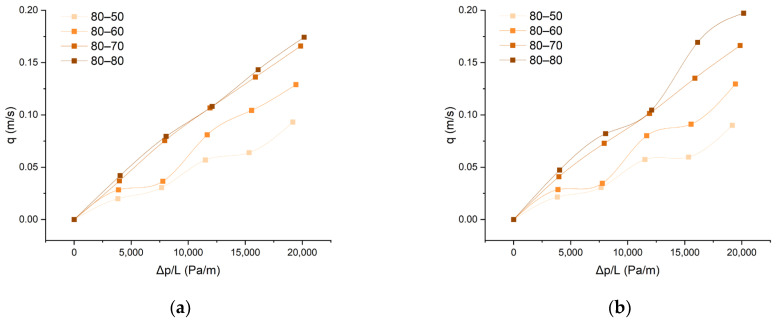
Non-Darcy air permeability fabrics’ behavior: (**a**) front (aramid) side of the woven fabric and (**b**) back (modacrylic) side of the woven fabric.

Samples exhibiting nonlinear air permeability behavior have been identified. From the graphs in Figure 8, it is evident that there is no linear relationship between flow rate and pressure drop. At certain pressures, the change in airflow velocity through the material is very small. This is due to differences in the structural characteristics of the two layers of the composite, which influence the size and stability of the vertical pores. These samples are the ones designated as 50-80, 80-50, and 80-60. In the case of the 80-80 sample, which is made from the finest yarns in the warp and weft (both layers), significant nonlinearity is observed upon direct contact with the airflow on the acrylic side. The cause of such nonlinear air permeability behavior could be attributed to the deformation of vertical pores in both layers. Under pressure, the sample undergoes compression, and the yarns have enough space for inter-structural displacements, resulting in changes in pore size and shape under pressure.

**Figure 9:**
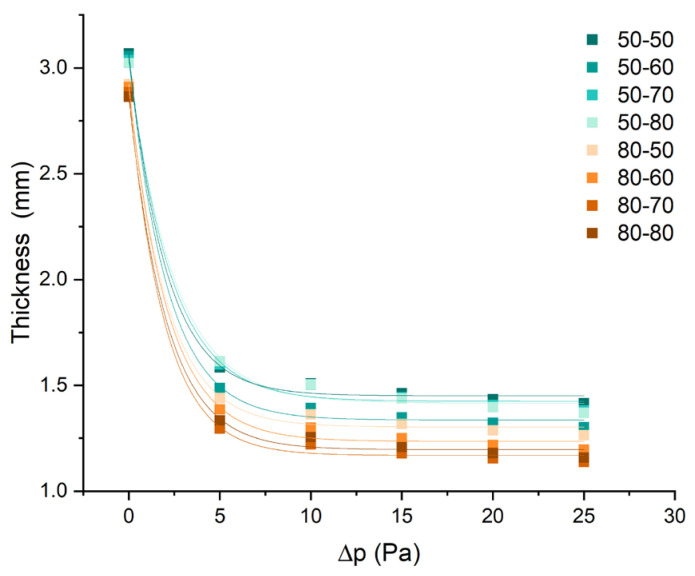
The change in fabric thickness at different pressure forces (Fabric Touch Tester results).

With the application of Darcy’s law, i.e., finding a Darcy’s constant implies a constant fabric architecture, however, woven fabrics have a certain compressive flexibility that depends on the yarn density and the weave and porosity. With increasing air pressures on the woven fabric surface, there is an evident non-linear decrease in thickness (Figure 9). This may lead to K, a dynamic value based on Darcy’s law, especially at the beginning of testing.

**Figure 10:**
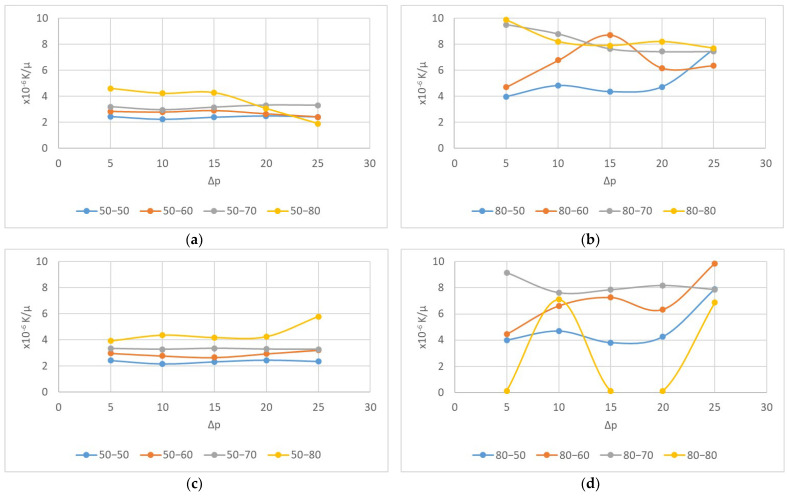
(**a**) Fabric front, warp: AR50; (**b**) fabric front, warp: AR80; (**c**) fabric back, warp: AR50; (**d**) fabric back, warp: AR80.

In Figure 10, the values of current slopes for samples with nonlinear air permeability are presented. This nonlinearity is not a result of reduced fabric thickness but rather the deformable internal structure of the two-layer woven fabrics. It is evident that fabric samples with a coarser fineness of the aramid weft exhibit more stable air permeability compared to those with a finer aramid weft. Generally, structures made from yarns with lower fineness values are more deformable, and the intensity of deformation increases with a higher proportion of coarser weft yarns.

**Figure 11:**
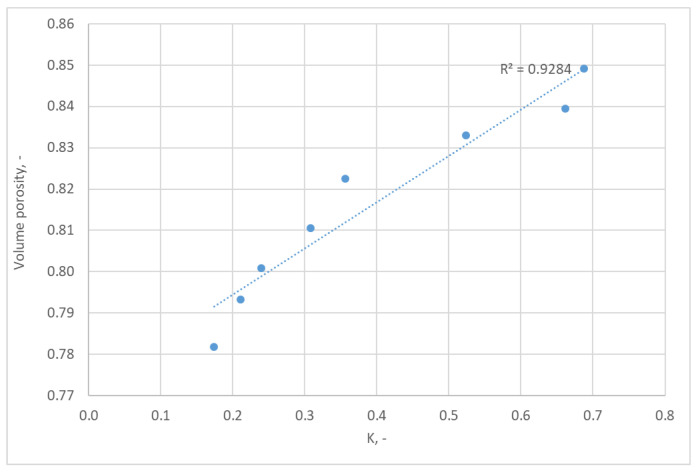
Relationship between volume porosity and Darcy’s constant K.

The ε/K graph (Figure 11) exhibits a deviation from linearity. Although the coefficient of determination is 0.93, the observed deviations could be due to the presence of ineffective porosity. Ineffective porosity refers to regions within the fabric structure that do not contribute significantly to the airflow. These regions may include areas with blocked or restricted airflow due to the woven fabric’s complex structure or the presence of certain fibers. These factors can lead to deviations from the expected linear relationship between porosity and air permeability.

## 6. Conclusions

The nonlinearity of air permeability in woven fabrics used for protective clothing can be attributed to the deformation of volume pores and changes in pore size and shape under pressure. As air pressure increases, the fabric experiences compression, which could cause the deformation of the pores within the fabric structure. This deformation affects the size and shape of the pores, resulting in nonlinear changes in air permeability. Furthermore, the relationship between fabric thickness and air pressure is also nonlinear. As the air pressure increases, the fabric thickness decreases in a nonlinear manner. This behavior indicates the compressive flexibility of the woven fabric, as it can undergo changes in thickness in response to applied pressure. The non-linear decrease in thickness suggests that the fabric structure allows for inter-structural displacements and adjustments, leading to changes in the fabric’s overall thickness under pressure. Regarding the yarn fineness, fabrics with a coarser fineness of the aramid weft exhibit more stable air permeability compared to those with a finer aramid weft. Coarser aramid weft yarns provide a more robust and consistent structure within the fabric, contributing to a more stable airflow and permeability.

Overall, the nonlinearity of air permeability in complex structured woven fabrics used for protective clothing is influenced by the deformation of volume pores, changes in pore size and shape under pressure, the compressive flexibility of the woven fabric, the yarn fineness, and the presence of ineffective porosity. Understanding these factors and their impact on air permeability can help in the design and development of more effective and reliable protective fabrics with controlled permeability properties. The application of Darcy’s law in assessing the breathability of woven fabrics used for thermal protection is significant, due to thermal comfort which is a property that is often neglected.

The study suggests that the application of Darcy’s law in complex fabric structures can be extended to all elastic woven fabrics with highly complex structures for various non-engineering purposes. This indicates the potential for further development and broader applications of Darcy’s law in analyzing the permeability of fabrics in diverse fields.

## Figures and Tables

**Table 1:** Declared parameters of the used yarns.

Designation	Composition	Tt (Tex)	T (Twist/m)
AR	95% M-aramid Conex NEO; 5% P-aramid Twaron	20 × 2	740 Z; 600 S
		17 × 2	840 Z; 660 S
		14 × 2	940 Z; 710 S
		12.5 × 2	1030 Z; 760 S
MC	45% cotton fiber (combed); 55% modacrylic fiber Sevel FRSA/L	20 × 2	970 Z; 670 S
		17 × 2	1000 Z; 720 S
		14 × 2	1170 Z; 800 S
		12.5 × 2	1200 Z; 760 S

**Table 2:** Description of the designed and woven fabric samples.

Sample	Warp	Tt (Tex)	Weft 1	Tt (Tex)	Weft 2	Tt (Tex)
50-50	AR	17 × 2	AR	20 × 2	MC	20 × 2
50-60	AR	17 × 2	AR	20 × 2	MC	17 × 2
50-70	AR	17 × 2	AR	20 × 2	MC	14 × 2
50-80	AR	17 × 2	AR	20 × 2	MC	12.5 × 2
80-50	AR	17 × 2	AR	12.5 × 2	MC	20 × 2
80-60	AR	17 × 2	AR	12.5 × 2	MC	17 × 2
80-70	AR	17 × 2	AR	12.5 × 2	MC	14 × 2
80-80	AR	17 × 2	AR	12.5 × 2	MC	12.5 × 2

**Table 3:** Basic parameters of the woven fabric samples.

Designation	Warp Density, Thread/cm	Weft Density, Thread/cm	Thickness, mm	Mass, g/m^2^
50-50	34	68	1.408	418
50-60	34	66	1.372	382
50-70	34	64	1.369	367
50-80	34	64	1.338	345
80-50	34	65	1.343	323
80-60	34	64	1.333	300
80-70	34	64	1.298	282
80-80	34	64	1.282	261

## Data Availability

Data available on request due to restrictions e.g., privacy or ethical. The data presented in this study are available on request from the corresponding author. The data are not publicly available. Access to the data is strictly limited to authorized personnel who have signed non-disclosure agreement within the project KK.01.2.1.02.0064 and are bound by strict confidentiality obligations.

## References

[1] Kiš A., Brnada S., Kovačević S. (2020). Influence of Fabric Weave on Thermal Radiation Resistance and Water Vapor Permeability. Polymers.

[2] Hursa Šajatović A., Dragčević Z., Pavlinić D.Z., Čupić M. (2016). Istraživanje stanja zaštitne odjeće za vatrogasce putem ankete. Tekstil.

[3] Perera Y.S., Muwanwella R.M.H.W., Fernando P.R., Fernando S.K., Jayawardana T.S.S. (2021). Evolution of 3D weaving and 3D woven fabric structures. Fash. Text..

[4] Galuszynskia S. (2014). Structure and Tightness of Woven Fabrics. Indian J. Text. Res..

[5] Havlová M. (2014). Model of Vertical Porosity Occurring in Woven Fabrics and its Effect on Air Permeability. Fibres Text. East. Eur..

[6] Elnashar E. (2005). Volume porosity and permeability in double-layer woven fabrics. Autex Res. J..

[7] Büyükbayraktar R., Okur A. (2012). Investigation of pore parameters of woven fabrics by theoretical and image analysis methods. J. Text. Inst..

[8] Dubrovski P.D. (2010). Woven fabrics and ultraviolet protection. Woven Fabric Engineering.

[9] Kalazić A., Brnada S., Kiš A. (2022). Thermal Protective Properties and Breathability of Multilayer Protective Woven Fabrics for Wildland Firefighting. Polymers.

[10] Gray W.G., O’Neill K. (1976). On the general equations for flow in porous media and their reduction to Darcy’s law. Water Resour. Res..

[11] Thusyanthan N.I., Madabhushi S.P. (2003). Scaling of seepage flow velocity in centrifuge models. Acta Gastroenterol. Latinoam..

[12] Govindarajan S.K. (2019). An overview on extension and limitations of macroscopic Darcy’s law for a single and multi-phase fluid flow through a porous medium. Int. J. Min. Sci..

[13] Dagan G. (1979). The generalization of Darcy’s law for nonuniform flows. Water Resour. Res..

[14] Van der Sluys L., Dierickx W. (1987). The applicability of Darcy’s law in determining the water permeability of geotextiles. Geotext. Geomembr..

[15] Patanaik A., Anandjiwala R. (2009). Some studies on water permeability of nonwoven fabrics. Text. Res. J..

[16] Bhattacharjee D., Ray A., Kothari V.K. (2004). Air and water permeability characteristics of nonwoven fabrics. Indian J. Fibre Text. Res..

[17] Chan A.W., Morgan R.J. (1993). Tow impregnation during resin transfer molding of bi-directional nonwoven fabrics. Polym. Compos..

[18] Hong Y.S., Wu C.S. (2011). Filtration behaviour of soil-nonwoven geotextile combinations subjected to various loads. Geotext. Geomembr..

[19] Turtoi P., Cicone T., Fatu A. (2017). Experimental and theoretical analysis of (water) permeability variation of nonwoven textiles subjected to compression. Mech. Ind..

[20] Rozy M.I., Ueda M., Fukasawa T., Ishigami T., Fukui K. (2020). Direct numerical simulation and experimental validation of flow resistivity of nonwoven fabric filter. AIChE J..

[21] Yang T., Hu L., Petrů M., Wang X., Xiong X., Yu D., Mishra R., Militký J. (2022). Determination of the permeability coefficient and airflow resistivity of nonwoven materials. Text. Res. J..

[22] Kulichenko A.V., Langenhove L.V. (1992). The resistance to flow transmission of porous materials. J. Text. Inst..

[23] Gooijer H., Warmoeskerken M.M., Groot Wassink J. (2003). Flow resistance of textile materials: Part I: Monofilament fabrics. Text. Res. J..

[24] Chen Z.R., Ye L., Lu M. (2010). Permeability predictions for woven fabric preforms. J. Compos. Mater..

[25] Gebart B.R. (1992). Permeability of unidirectional reinforcements for RTM. J. Compos. Mater..

[26] Adams K.L., Miller B., Rebenfeld L. (1986). Forced in-plane flow of an epoxy resin in fibrous networks. Polym. Eng. Sci..

[27] Dimitrovski K., Zupin Ž., Kostajnšek K., Branca E. (2017). Use of air permeability for determination of equivalent average pore diameter in woven fabrics. IOP Conf. Ser. Mater. Sci. Eng..

[28] Hu J. (2008). 3-D fibrous assemblies: Properties, applications and modelling of three-dimensional textile structures. Permeability of Multilayer Woven Fabrics.

[29] Alotaibi H., Jabbari M., Abeykoon C., Soutis C. (2022). Numerical Investigation of Multi-scale Characteristics of Single and Multi-layered Woven Structures. Appl. Compos. Mater..

[30] Thomas S., Bongiovanni C., Nutt S.R. (2008). In situ estimation of through-thickness resin flow using ultrasound. Compos. Sci. Technol..

[31] Karaki M. (2017). Experimental Study and Modelling of Permeability of Engineering Textiles Used in Composite Materials. Ph.D. Thesis.

[32] Xiao X. (2012). Modeling the Structure-Permeability Relationship for Woven Fabrics. Ph.D. Thesis.

[33] Xiao X., Long A., Qian K., Zeng X., Hua T. (2017). Through-thickness permeability of woven fabric under increasing air pressure: Theoretical framework and simulation. Text. Res. J..

[34] Khan M.A.A. (2021). In-Plane Permeability Measurement of Biaxial Woven Fabrics by 2D-Radial Flow Method. Sci. Eng. Compos. Mater..

[35] Dei Sommi A., Lionetto F., Maffezzoli A. (2023). An Overview of the Measurement of Permeability of Composite Reinforcements. Polymers.

[36] Atangana A. (2017). Fractional Operators with Constant and Variable Order with Application to Geo-Hydrology.

[37] Badrov T., Schwarz I., Kovačević S. (2022). Multifunctionality of Thermal Protective Layer Interchanging Double Cloth Conditioned by Influential Parameters. Polymers.

